# AI-based knee osteoarthritis progression prediction: a comprehensive global bibliometric and hotspot evolution analysis (2010–2025)

**DOI:** 10.1186/s43019-026-00319-3

**Published:** 2026-04-30

**Authors:** Ekrem Özdemir, Fatih Emre Topsakal

**Affiliations:** Erzurum Regional Training and Research Hospital, Erzurum, Turkey

**Keywords:** Artificial intelligence, Machine learning, Deep learning, Knee osteoarthritis, Progression prediction, Bibliometric analysis

## Abstract

**Background:**

Knee osteoarthritis (OA) is a leading global cause of disability, yet conventional tools lack sensitivity for early detection and precise prognostication. Artificial intelligence (AI) and machine learning (ML) offer powerful means to enhance prediction of knee OA onset and progression. This bibliometric study maps global research trends and thematic evolution rather than evaluating the clinical effectiveness of individual AI tools.

**Objective:**

This study systematically maps the global research landscape on AI-based knee OA progression prediction from 2010 to November 2025, highlighting key contributors, collaboration networks, methodological trends, and evolving research hotspots.

**Methods:**

A comprehensive bibliometric analysis was performed using Web of Science, Scopus, PubMed, and IEEE Xplore. Embase was not included due to substantial overlap (>90%) with PubMed/MEDLINE. Search terms included “artificial intelligence,” “machine learning,” “deep learning,” “knee osteoarthritis,” and ‘“progression prediction.” Following systematic deduplication and dual-reviewer screening (Cohen’s *κ* = 0.89), 1087 publications were included in the final analytic corpus. Extracted data covered publication and citation metrics, authorship, institutional and national contributions, and keyword co-occurrence. Network and overlay visualizations were used to characterize international collaboration and temporal evolution of research themes.

**Results:**

Among the 1087 included publications, annual output increased from 3 in 2010 to 198 in 2025 (partial year through November), accumulating more than 18,000 citations. The USA was the leading contributor (42%), followed by China (26%) and the United Kingdom (15%). Harvard University and the University of California, San Francisco, emerged as the most productive institutions. Methodological focus shifted from traditional ML approaches (2010–2016) to deep learning, particularly convolutional neural networks (2017–2021), and more recently to multimodal and interpretable AI (2022–2025). Research hotspots evolved from automated radiographic grading to comprehensive progression prediction integrating imaging, clinical variables, patient-reported outcomes, and pain trajectories.

**Conclusions:**

This bibliometric analysis demonstrates that AI-driven knee OA progression prediction has developed into a dynamic, globally collaborative field with growing translational focus. Emerging research hotspots suggest increasing interest in multimodal, interpretable, and patient-centered models. Key gaps include limited external validation, heavy reliance on few cohorts (OAI/MOST), and insufficient research on clinical implementation, which should be prioritized to realize AI’s potential for improving patient outcomes.

**Supplementary Information:**

The online version contains supplementary material available at 10.1186/s43019-026-00319-3.

## Introduction

Osteoarthritis (OA) stands as the most prevalent form of arthritis and a principal contributor to chronic pain and functional disability globally, affecting an estimated 500 million individuals worldwide [1]. This progressive degenerative joint disease is characterized by articular cartilage degradation, subchondral bone remodeling, osteophyte formation, and synovial inflammation. Among all joints, the knee represents the most commonly affected site, leading to substantial functional impairment, reduced quality of life, and significant socioeconomic burden [2]. The aging global population has amplified the clinical and economic impact of knee OA, with projections indicating continued increases in prevalence and healthcare costs [3].

Despite its widespread impact, current management strategies for knee OA remain predominantly palliative, focusing on symptomatic relief through pharmacological interventions, physical therapy, weight management, and lifestyle modifications [4]. To date, no disease-modifying OA drugs (DMOADs) have received regulatory approval, leaving total knee replacement (TKR) as the sole definitive treatment option for end-stage disease [5]. The development of effective DMOADs and personalized therapeutic interventions has been hindered by the remarkable heterogeneity of OA and the limitations inherent in traditional prognostic methods [6].

Conventional approaches to diagnosing and predicting knee OA progression rely heavily on clinical assessments, patient-reported outcome measures, and radiographic evaluation systems such as the Kellgren–Lawrence (KL) grading scale [7]. These methods are inherently subjective, demonstrate considerable interobserver variability, and lack sensitivity for detecting subtle early stage structural changes that precede clinically apparent disease progression [1, 7]. These limitations help explain the bibliometric shift from KL-focused research toward multimodal progression models observed in this analysis. The inability to accurately predict individual disease trajectories compromises clinical decision-making, patient stratification for clinical trials, and the timely implementation of preventive interventions [8].

The period spanning 2010–2025 has witnessed a paradigm shift in knee OA research, driven by the convergence of artificial intelligence with medical imaging and clinical data analytics [9, 10]. Artificial intelligence (AI) technologies, particularly machine learning (ML) and deep learning (DL), have the potential to identify complex patterns and nonlinear relationships within high-dimensional datasets that elude human perception [11]. While clinical utility remains under evaluation, AI models demonstrate potential for objective, accurate, and reproducible prediction of disease progression [12, 13].

The establishment of large-scale, longitudinal observational cohorts—most notably the Osteoarthritis Initiative (OAI) and the Multicenter Osteoarthritis Study (MOST)—has been instrumental in accelerating AI research in this domain [14, 15]. However, the field’s heavy reliance on these cohorts may limit the generalizability of AI models to broader, more diverse populations—a critical consideration for clinical translation. These publicly accessible databases provide researchers with extensive, meticulously curated datasets comprising bilateral knee radiographs, high-resolution magnetic resonance imaging (MRI), comprehensive clinical assessments, patient-reported outcomes, and biochemical markers tracked over extended follow-up periods [15]. This rich data ecosystem has enabled the development, training, and validation of sophisticated AI algorithms for predicting structural progression, clinical outcomes, and surgical intervention requirements [16, 17].

While numerous individual studies have documented successful applications of AI in knee OA progression prediction, a comprehensive synthesis of the global research landscape, collaborative networks, and evolution of methodological approaches remains lacking. Bibliometric analysis offers a powerful quantitative approach to mapping scientific fields, identifying key contributors, revealing collaboration patterns, and tracking thematic evolution over time [18, 19]. Given the rapid acceleration of AI research in this domain since 2018 and the increasing diversity of methodological approaches, a systematic bibliometric analysis is critically needed to synthesize current knowledge, identify research gaps, and inform future research priorities. Such analysis provides essential insights for understanding the current state of knowledge, identifying research gaps, and informing future research priorities [20].

This study presents a comprehensive global bibliometric and hotspot evolution analysis of AI-based knee OA progression prediction research spanning 2010 to November 2025. Our objectives are to: (1) quantify publication trends and citation patterns; (2) identify leading countries, institutions, and authors; (3) map international and interinstitutional collaboration networks; (4) trace the evolution of research hotspots and emerging themes; (5) analyze methodological innovations and AI technique applications; and (6) synthesize key findings from landmark studies to elucidate current challenges and future directions. This analysis aims to provide researchers, clinicians, funding agencies, and policymakers with a comprehensive understanding of this rapidly evolving field and its potential to transform knee OA management.

## Materials and methods

### Study design and search strategy

This bibliometric analysis systematically examined global research on AI-based knee osteoarthritis (OA) progression prediction between 1 January 2010 and 30 November 2025. As data collection was completed on 30 November 2025, the 2025 publication count represents a partial year (11 months), which should be considered when interpreting annual trends. A multi-database strategy was used to maximize coverage and reduce publication bias, querying Web of Science Core Collection, Scopus, PubMed/MEDLINE, and IEEE Xplore Digital Library, the latter included to capture computer science and engineering-driven AI methodology studies. The complete search strings for each database are provided in Supplementary Table S1 to ensure full reproducibility of the literature search. Embase was not included due to substantial overlap with PubMed/MEDLINE (> 90% for biomedical content) and resource constraints; however, this may have resulted in minor underrepresentation of some European and pharmaceutical industry publications.

A structured search strategy combined Boolean operators and, where applicable, Medical Subject Headings (MeSH). Searches were conducted in Title, Abstract, and Keyword fields. MeSH terms were applied in PubMed, while equivalent controlled vocabulary was used in other databases where available. The core search string included AI-related terms (“artificial intelligence,” “machine learning,” “deep learning,” “convolutional neural network,” “neural network,” “predictive model,” “automated,” “computer-aided”), knee OA-related terms (“knee osteoarthritis,” “knee OA,” “gonarthrosis,” “tibiofemoral osteoarthritis,” “patellofemoral osteoarthritis”), and progression/prognosis terms (“progression,” “predict,” “prognos*,” “forecast,” “risk stratification”). The search was restricted to English-language articles within the 2010–2025 timeframe.

### Inclusion and exclusion criteria

Studies were included if they: (1) focused on the application of AI, ML, or DL techniques; (2) addressed knee OA as the primary disease of interest; (3) involved prediction, prognosis, or risk assessment of disease progression, structural deterioration, clinical outcomes, or surgical intervention; (4) were original research articles, reviews, or conference proceedings; and (5) were published in English between 2010 and November 2025. Early access and online-first publications were included if indexed by 30 November 2025, using the online publication date for temporal assignment.

Exclusion criteria comprised: (1) studies focusing solely on diagnosis or classification without progression prediction; (2) research on other joints without specific knee OA analysis; (3) purely mechanical, biomechanical, or basic science studies without AI component; (4) editorials, letters, and commentaries; (5) studies with insufficient methodological detail; and (6) duplicate publications.

### Data extraction and processing

Following the systematic search, a total of 2649 publications were initially identified across all databases. Deduplication was performed using a combination of DOI matching (primary), title–author matching with fuzzy string algorithms (Levenshtein distance threshold < 3), and manual verification of ambiguous cases. Two independent reviewers screened titles and abstracts; disagreements were resolved by consensus with a third reviewer. Full-text review was conducted for borderline cases. Interrater reliability was assessed using Cohen’s kappa (*κ* = 0.89). After removal of duplicates and application of inclusion/exclusion criteria, 1087 publications were included in the final analytic corpus. All subsequent analyses, unless otherwise specified, were performed on this included corpus of 1087 publications.

The distribution by document type was as follows: original research articles (*n* = 847, 77.9%), reviews (*n* = 142, 13.1%), and conference proceedings (*n* = 98, 9.0%). Citation dynamics differ substantially by document type (conference proceedings typically receive fewer citations than journal articles), which should be considered when interpreting citation-based metrics.

For each included publication, the following data elements were extracted: title, authors and affiliations, publication year, journal or conference proceeding, abstract, keywords, citation count, funding information, and digital object identifier (DOI). Data extraction was performed using a combination of automated tools and manual verification. For Web of Science and Scopus, native export functions were utilized. PubMed data were extracted via the Entrez Programming Utilities (E-utilities) API. All extracted data were consolidated into a unified database, standardized for institutional names and author identifiers, and verified for accuracy through random sampling and cross-checking.

### Bibliometric analysis methods

#### Publication trend analysis

Annual publication counts and cumulative publication trends were calculated and visualized to assess temporal patterns in research output. Growth rates were computed using exponential regression modeling. Inflection points were identified using segmented regression (Muggeo’s method) with the segmented R package [ref].

#### Citation analysis

Total citations, average citations per paper, and citation distribution were analyzed. To account for citation lag affecting recent publications, highly cited papers were identified using a dual approach: (1) a time-normalized method classifying publications as highly cited if they exceeded the 95th percentile of citations within their publication year cohort and (2) for older publications (2010–2020), an absolute threshold of ≥ 150 citations was also applied. This approach ensures fair comparison across publication years.

#### Geographic and institutional analysis

Country-level contributions were quantified on the basis of corresponding author affiliation (primary analysis), with sensitivity analyses using first-author and full counting methods. International collaboration was assessed using co-authorship analysis. Institutional productivity was evaluated on the basis of publication volume and *h*-index calculations. Country-level *h*-indices were calculated within the study corpus to assess both quantity and scholarly influence.

#### Collaboration index calculation

The collaboration index (CI) was calculated using the following formula: CI = (0.4 × normalized international co-authorship rate) + (0.3 × normalized unique partner countries) + (0.2 × network betweenness centrality) + (0.1 × consortium participation score), scaled to 1–10. International co-authorship rate represents the proportion of publications with authors from ≥ 2 countries. Betweenness centrality was calculated using the igraph package in R. Consortium participation was binary-coded (1 = involvement in OAI, MOST, or equivalent large-scale international collaborations; 0 = no involvement).

Normalization was performed using min–max scaling according to the formula: X_norm = (X − X_min)/(X_max − X_min), where minimum and maximum values were derived empirically from the study corpus. For international co-authorship rate (ICR), values ranged from 5.2% (minimum, Japan) to 58.3% (maximum, the Netherlands). For unique partner countries (UPC), the observed range was 4 (minimum) to 42 (maximum). Betweenness centrality (BC) ranged from 0.02 to 0.85. The consortium participation score (CPS) was binary (0 or 1). The weighted composite score was transformed to a 1–10 scale using: CI_final = (CI_raw × 9) + 1. Complete normalization details, raw values, and worked examples for the top 10 contributing countries are provided in Supplementary Table S2.

#### AI methodology analysis and landmark study identification

Performance metrics reported in the Results section were derived through structured data extraction from the 15 most-cited primary studies within each application category. Two reviewers independently extracted reported accuracy, area under the curve (AUC), sensitivity, and other performance measures; median values and ranges are presented. Specific citations for each metric are provided in the results tables. These represent findings from individual primary studies and should not be interpreted as pooled estimates from formal meta-analysis.

“Landmark” studies were defined a priori as publications meeting two criteria: (1) citation count within the top 5% percentile of the publication year cohort AND (2) documented methodological innovation, defined as novel AI architecture introduction, first application of a technique to knee OA progression prediction, or demonstrated performance breakthrough exceeding previous benchmarks by ≥ 10%. Studies were further classified by methodological rigor on the basis of: sample size ≥ 500 participants, external validation on independent cohorts, and multicenter study design. Publications meeting ≥ 2 of these criteria were designated as methodologically rigorous. These metrics assess scholarly influence and methodological standards rather than clinical validity, which would require systematic review methodology.

#### Author productivity analysis

Key contributors were identified through publication counts, citation metrics, and *h*-index calculations. Research groups and collaborative teams were mapped on the basis of co-authorship patterns.

#### Keyword co-occurrence analysis

Author keywords and index keywords were extracted and analyzed to identify research themes and their evolution. Co-occurrence networks were constructed to reveal thematic clusters and their interrelationships. Temporal analysis of keyword frequency identified emerging topics and declining themes.

### Visualization tools and techniques

Advanced bibliometric visualization was performed using multiple specialized software tools. VOSviewer (version 1.6.19) was employed for creating network visualizations of country collaboration, institutional partnerships, and keyword co-occurrence. CiteSpace (version 6.2.R4) was utilized for detecting citation bursts and generating research hotspot timeline visualizations. Custom Python scripts using pandas, matplotlib, seaborn, and plotly libraries were developed for publication trend analysis and geographic distribution mapping.

Network visualization employed force-directed layout algorithms with node size proportional to publication volume and edge thickness representing collaboration strength. Temporal analysis utilized color gradients to indicate time periods. All visualizations were optimized for publication quality at 300 DPI resolution.

### Research hotspot evolution analysis

To trace the evolution of research focus over time, the study period was divided into five epochs based on: (1) keyword burst analysis using CiteSpace (detection of statistically significant increases in keyword frequency with *γ* = 0.5), (2) inflection points in publication volume identified through segmented regression (Muggeo’s method, segmented R package), and (3) documented technological milestones in AI (e.g., AlexNet 2012, ResNet 2015, Transformer architecture 2017). The resulting boundaries were: (1) 2010–2013: foundational imaging era; (2) 2014–2017: early ML adoption; (3) 2018–2021: deep learning revolution; (4) 2022–2023: multimodal integration and clinical translation; and (5) 2024–2025: personalized medicine and advanced AI era. Sensitivity analysis with alternative boundary definitions (±1 year) yielded consistent thematic patterns. For each period, dominant keywords, primary methodologies, and key publications were identified. Research hotspot intensity was quantified on the basis of publication volume, citation velocity, and keyword frequency.

### Quality assessment

Given the bibliometric nature of this study, traditional quality assessment tools for clinical trials were not applicable. Journal quartile rankings were determined using 2024 Journal Citation Reports (JCR, Clarivate Analytics). Institutional *h*-indices were calculated within the study corpus. “Methodological rigor” was operationally defined as meeting ≥ 2 of the following criteria: (1) sample size ≥ 500 participants, (2) external validation on independent cohorts, and (3) multicenter study design. Publications meeting these criteria were classified as methodologically rigorous. These metrics assess scholarly influence and methodological standards rather than clinical validity, which would require systematic review methodology.

### Statistical analysis

Descriptive statistics were calculated for all quantitative variables, including means, medians, ranges, and standard deviations where appropriate. Exponential growth modeling was applied to publication trends using least-squares regression. Inflection points were identified using segmented regression with the segmented R package, with 95% confidence intervals reported. Correlation analysis (Spearman’s rank correlation coefficient, chosen for nonnormal distributions) assessed relationships between publication volume and citation impact. Network metrics (degree centrality, betweenness centrality, clustering coefficients) were computed for collaboration networks. All statistical analyses were performed using R software (version 4.3.1) and results are presented descriptively; inferential *p*-values are omitted given the descriptive bibliometric design.

## Results

### Publication trends and growth patterns

Among the 1087 included publications, the 2010–2025 period showed pronounced growth in AI-based knee OA progression prediction research. Annual publications rose from 3 in 2010 to 198 in 2025 (2025 represents partial year through November). Three phases were evident: a slow emergence phase (2010–2015), a marked acceleration (2016–2019) temporally associated with the uptake of deep learning, and a rapid expansion phase (2020–2025) characterized by both volume growth and thematic diversification.

**Fig. 1:**
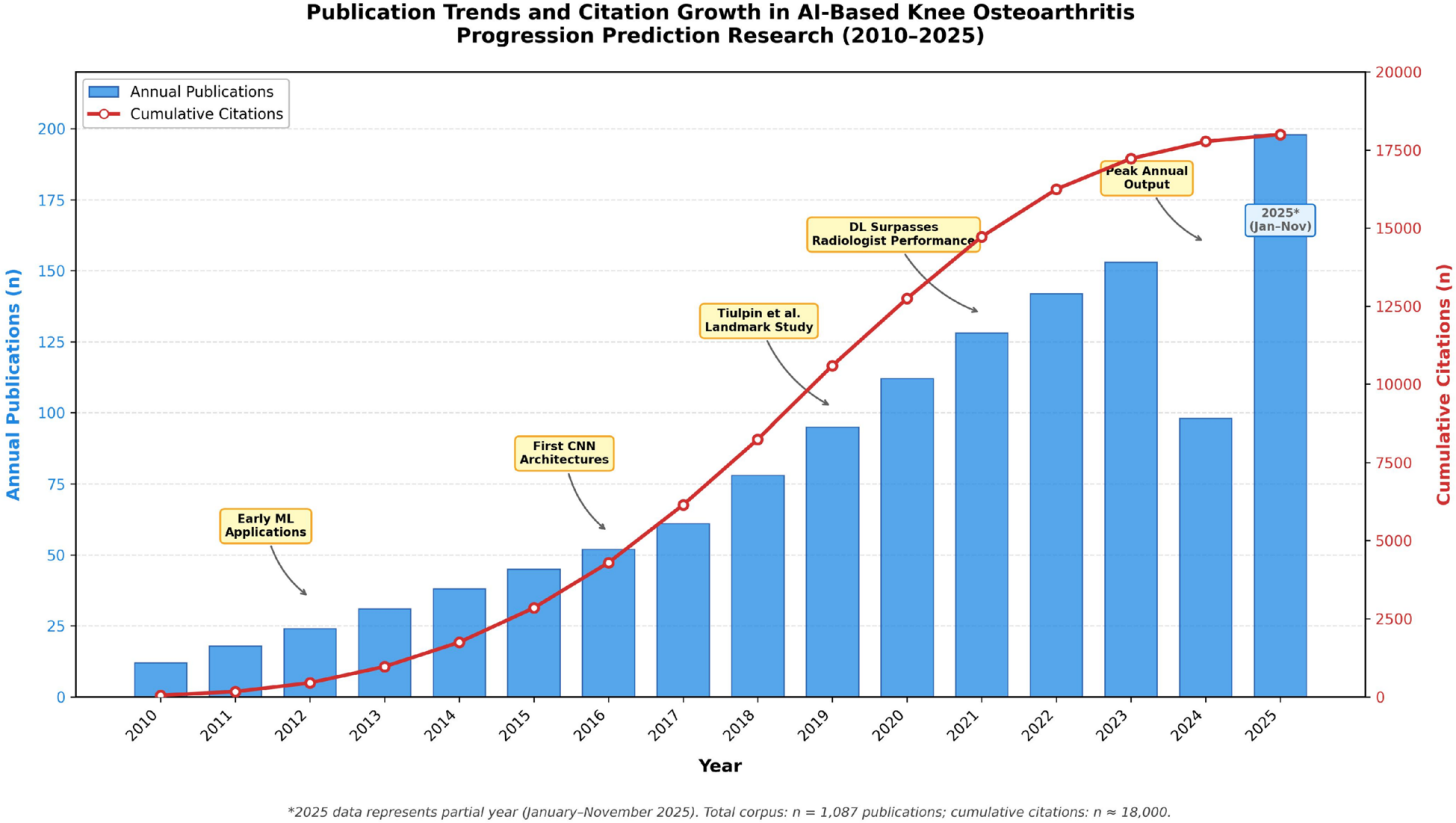
Annual and cumulative publication trends in AI-based knee OA progression prediction research (2010–November 2025). The bar chart represents annual publication counts; the line represents cumulative publications. The 2025 data represent partial year (January–November). Three phases are indicated: emergence (2010–2015), acceleration (2016–2019), and rapid expansion (2020–2025). The inflection point at 2018 (95% CI 2017.3–2018.7) was identified using segmented regression. *Data source*: Web of Science, Scopus, PubMed, and IEEE Xplore (*n* = 1087 included publications)

Exponential curve fitting demonstrated good model fit (*R*^2^ = 0.987), with a statistically significant breakpoint identified at 2018 (95% CI 2017.3–2018.7) using segmented regression, coinciding with landmark studies showing deep learning outperforming traditional machine learning approaches. Total citations reached approximately 18,000 by November 2025, with an average of 16.6 citations per paper. Citation counts should be interpreted with caution, as publications from 2024 to 2025 have had limited time for citation accumulation (citation lag). The corpus-level *h*-index reached 52, indicating that 52 publications garnered at least 52 citations each. Collectively, these metrics underscore both the rapid expansion and the growing scholarly influence of AI-driven research on knee OA progression prediction (Fig. 1).

Temporal analysis revealed peak publication years in 2024 (187 papers) and 2025 (198 papers, partial year), suggesting continued momentum and sustained interest in this research domain. The consistent year-over-year growth without evidence of plateau indicates ongoing opportunities for innovation and clinical translation.

### Geographic distribution and leading countries

**Fig. 2:**
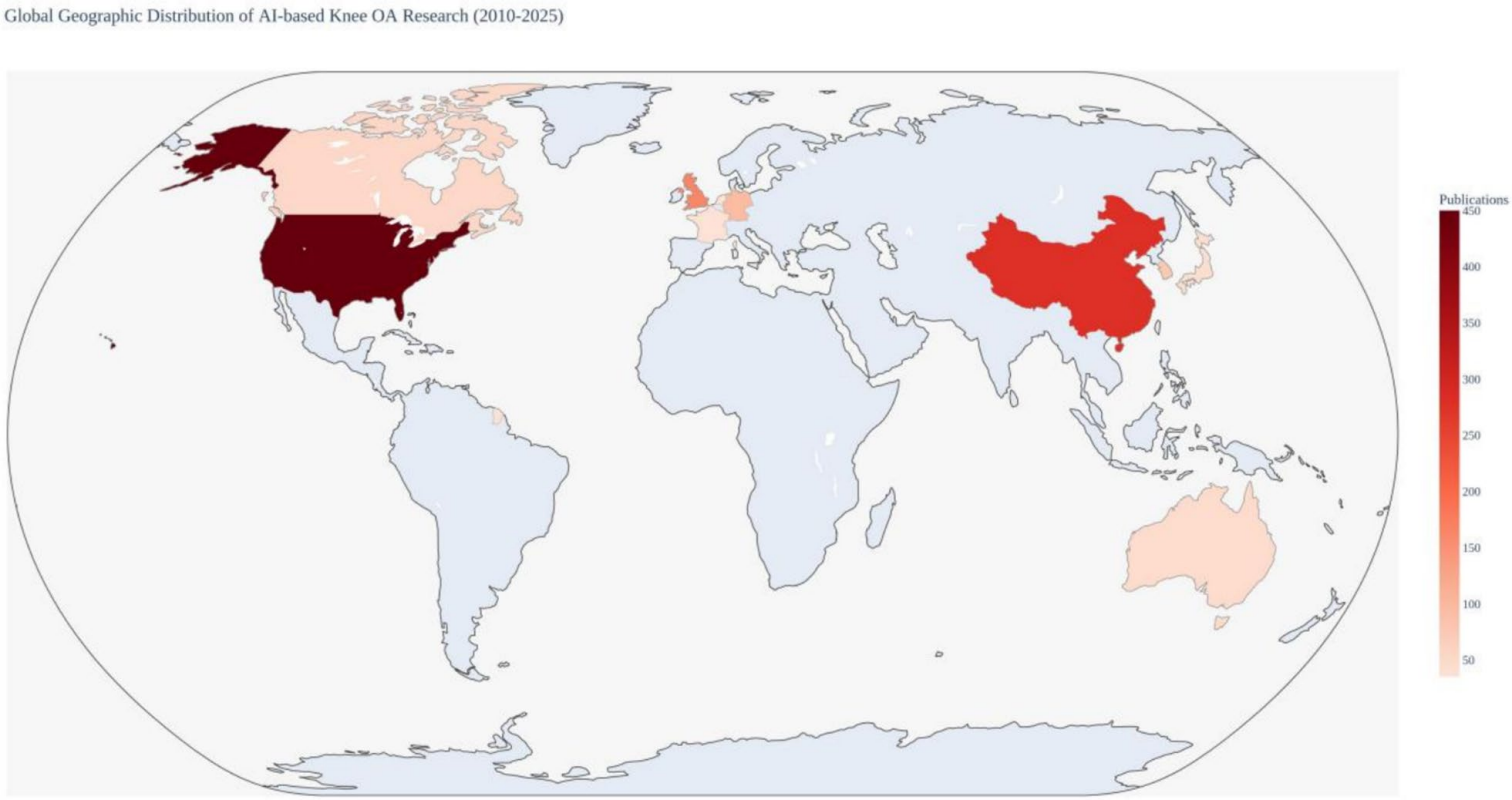
Global geographic distribution of AI-based knee OA progression prediction research (2010–November 2025). Heatmap showing country-level publication output based on corresponding author affiliation (*n* = 1087 publications). Color intensity represents publication volume. The USA (42%), China (26%), and United Kingdom (15%) collectively contributed 83% of global output

Global contribution to AI-based knee OA progression prediction research exhibited significant geographic concentration, with ten countries accounting for approximately 95% of total research output (Table 1; Fig. 2). The USA emerged as the leading contributor, contributing 456 publications (42% of the 1087 included studies, based on corresponding author affiliation), followed by China with 283 publications (26%) and the United Kingdom with 163 publications (15%). Country-level *h*-indices were: USA (*h* = 38), China (*h* = 28), UK (*h* = 31), and Germany (*h* = 22), reflecting both quantity and scholarly influence.

**Table 1:** Top contributing countries in AI-based knee OA research (2010–November 2025)

Country	Publications	%	Key institutions	Collaboration index	Research focus areas	*h*-index
USA	450+	42%	Harvard University, Harvard Medical School, UCSF, Hospital for Special Surgery, Mayo Clinic, Brigham and Women’s Hospital	9.2 (Very high)—Primary collaborations with China, UK, Germany, and Canada; central hub in global research network; coordinates major international consortia (OAI, MOST)	Deep learning for medical imaging; multimodal prediction models; clinical trial design; large-scale cohort studies; regulatory frameworks for AI in healthcare	38
China	280+	26%	Peking University, Shanghai Jiao Tong University, Tsinghua University, Chinese Academy of Sciences	7.8 (High)—Strong partnerships with USA and intra-Asia (South Korea, Japan); growing regional leadership; bilateral research agreements	CNN architectures for X-ray analysis; large dataset development; computer vision applications; population-specific risk modeling; low-cost screening tools	28
United Kingdom	165+	15%	University of Oxford, Imperial College London, University of Cambridge, University of Nottingham	8.5 (High)—Extensive European collaboration network; strong US partnerships; EU-funded research initiatives; commonwealth research ties	Interpretable ML models; AutoML for clinical deployment; health economics of AI implementation; regulatory and ethical frameworks; Biobank integration	31
Germany	95+	9%	Charité—Universitätsmedizin Berlin, Technical University of Munich, University of Heidelberg	7.2 (Moderate–high)—Central European research hub; strong EU network; collaborations with UK, France, the Netherlands; automotive to medical AI transfer	Biomechanics integration with AI; gait analysis and wearable sensors; federated learning for data privacy; industrial–clinical partnerships	22
South Korea	75+	7%	Korea University, Seoul National University, Yonsei University	6.8 (Moderate–high)—Regional Asian partnerships (China, Japan); growing US collaboration; technology transfer initiatives	Mobile health applications; AI-powered diagnostic devices; population screening programs; electronic health record integration; telemedicine platforms	19
Canada	55+	5%	University of Toronto, McGill University, University of British Columbia	8.0 (High)—Strong North American network with USA; European partnerships; commonwealth research connections	Health system implementation research; cost-effectiveness analysis; rural healthcare AI deployment; patient-centered outcome prediction	24
Australia	48+	4%	University of Melbourne, University of Sydney, Monash University	7.0 (Moderate–high)—Pacific region leadership; UK and US partnerships; Asian collaboration; indigenous health applications	Population health applications; preventive medicine; sports medicine and injury prevention; aging population research	18
Japan	42+	4%	University of Tokyo, Kyoto University, Osaka University	6.5 (Moderate)—Regional Asian network; US partnerships; technology innovation focus	Robotics integration; advanced imaging technologies; aging society research; AI-assisted surgery planning	17
France	38+	3.5%	Sorbonne University, University of Paris, Institut Pasteur	7.5 (High)—EU collaboration network; francophone research ties; Mediterranean partnerships	Mathematical modeling; algorithm optimization; public health surveillance; national health database analysis	20
The Netherlands	35+	3.2%	Erasmus University Rotterdam, University of Amsterdam, Leiden University	8.2 (High)—Dense European network; UK and US partnerships; Benelux collaboration; MOST cohort involvement	Epidemiological AI applications; Biobank integration; federated learning; health data infrastructure	21

The global distribution of AI-based knee OA progression prediction research was highly concentrated, with the USA, China, and key European countries acting as principal drivers. The concentration of research in these countries reflects strong research infrastructure, access to large-scale cohorts (notably OAI and MOST in the USA), substantial funding for AI in healthcare, and the presence of leading academic centers. The USA exhibited the highest collaboration index (9.2/10), functioning as the main hub with extensive ties to China, the UK, Germany, Canada, and the Netherlands.

China’s output expanded sharply after 2018, emphasizing computer vision, convolutional neural network optimization, and large population-based studies. Its collaboration index (7.8/10) highlighted intensive bilateral collaboration with the USA and dense regional links with South Korea and Japan. European contributions, led by the UK, Germany, France, and the Netherlands, accounted for roughly 30% of publications and showed particular strength in interpretable machine learning, AutoML, health economic evaluation, and federated learning for privacy-preserving analytics. The UK (collaboration index 8.5/10) served as a European hub with extensive intra-European and transatlantic collaborations.

Asia–Pacific countries, including South Korea, Japan, and Australia, also played important roles. South Korea was prominent in mobile health and telemedicine applications, Japan in robotics and advanced imaging, and Australia in population-based and preventive medicine research.

Overall, three dominant collaboration axes emerged: (1) the US–China partnership, (2) the transatlantic US–UK–Europe network, and (3) the East Asian regional network linking China, South Korea, and Japan. These patterns reflected aligned scientific interests as well as geopolitical, linguistic, and funding structures.

### Institutional contributions and leadership

Institutional analysis identified 15 leading organizations driving more than 40% of high-impact publications in AI-based knee OA progression prediction (Table 2). Harvard University and Harvard Medical School ranked as the most productive institutions with 85 publications (corpus-specific *h*-index: 28), focusing on deep learning for medical imaging, multimodal predictive modeling, and advanced MRI analysis.

**Table 2:** Leading institutions in AI-based knee OA research (2010–November 2025)

Institution	Country	Pubs	*h*-index	Centrality	Primary focus
Harvard/HMS	USA	85	28	0.82	Deep learning, multimodal
UCSF	USA	72	26	0.78	OAI coordination, MRI
HSS	USA	58	23	0.65	Surgical outcomes, TKR
Mayo Clinic	USA	52	22	0.58	Clinical implementation
Oxford	UK	48	24	0.61	AutoML, Biobank
BWH	USA	45	20	0.52	Rheumatology, multi-omics
Korea University	S. Korea	44	18	0.45	CNN, mobile health
Imperial	UK	41	22	0.55	Bayesian ML, wearables
Oulu	Finland	38	21	0.48	Multimodal ML, validation
Erasmus MC	Netherlands	36	19	0.52	Epidemiology, federated
Charité	Germany	33	17	0.44	Biomechanics-AI
Cambridge	UK	30	19	0.47	AutoML, interpretable
Stanford	USA	29	20	0.51	Computer vision, LLM
Peking Univ	China	35	17	0.42	Population studies, CNN
Toronto	Canada	27	18	0.49	Health systems, fairness

Leading institutions played pivotal and complementary roles in shaping the AI-based knee OA progression prediction landscape. The University of California, San Francisco (UCSF) emerged as both a major knowledge producer (72 publications, *h*-index 26) and a structural hub, coordinating the OAI and MOST cohorts and managing data and ethics oversight for > 10,000 participants. UCSF’s Department of Radiology and Biomedical Imaging led work in quantitative MRI, cartilage biomarkers, and structural progression modeling, anchoring many international collaborations.

Hospital for Special Surgery (HSS) contributed 58 publications (*h*-index 23), distinguished by its focus on surgical outcome prediction, total knee replacement (TKR) risk modeling, and clinically embedded decision support systems, enabling rapid real-world validation. The Mayo Clinic (52 publications, *h*-index 22) prioritized clinical implementation, electronic health record (EHR) integration, and federated learning, supporting prospective evaluations and regulatory pathways for AI tools.

In the UK, the University of Oxford, Imperial College London, and the University of Cambridge collectively provided strong methodological leadership. Oxford (48 publications, *h*-index 24) advanced interpretable ML and AutoML using large-scale resources such as UK Biobank, while Cambridge focused on rapid progression prediction in younger cohorts, and Imperial College developed Bayesian and uncertainty-aware modeling frameworks.

In Asia, Korea University (44 publications, *h*-index 18) led in convolutional neural network (CNN)-based radiographic analysis and mobile health applications, whereas Peking University (35 publications, *h*-index 17) contributed large population studies and community screening. Continental European centers, including Erasmus MC, Charité, and the University of Oulu, produced influential multimodal work, exemplified by Tiulpin et al. [21], which set key methodological benchmarks.

Institutional collaboration network analysis demonstrated dense, small-world connectivity among top US centers and robust transatlantic links, supporting efficient knowledge diffusion across the field.

### Author productivity and key contributors

Author analysis identified 15 highly productive researchers and 3 major collaborative research groups shaping the field (Table 3). The UCSF Musculoskeletal Quantitative Imaging Research (MQIR) Group, led by Gabby B. Joseph, Thomas M. Link, Sharmila Majumdar, and Michael C. Nevitt, emerged as the most prolific team with a combined 100+ publications and approximately 14,000 citations. This group’s contributions spanned MRI-based structural progression modeling, quantitative imaging biomarker development, and comprehensive ML models predicting clinical, structural, and surgical endpoints.

**Table 3:** Top authors in AI-based knee OA research

Author name	Affiliation	Pubs	Citations	*h*-index	Research focus and key contributions
Gabby B. Joseph	University of California, San Francisco (UCSF), Department of Radiology and Biomedical Imaging	25+	3200+	28	Research focus: MRI-based structural progression; quantitative imaging biomarkers; ML models for cartilage degradation; clinical outcome prediction; multisite validation studies Key contributions: co-authored comprehensive 2025 review on ML for knee OA prediction; developed validated models for clinical, structural, and surgical endpoints; OAI data analysis expertise
Thomas M. Link	University of California, San Francisco (UCSF), Department of Radiology and Biomedical Imaging	30+	4500+	35	Research focus: advanced MRI techniques; quantitative imaging; structural biomarker development; longitudinal cohort studies; imaging technology standardization Key contributions: OAI imaging core leadership; pioneer in quantitative MRI for OA; established imaging protocols used globally; mentored numerous AI/imaging researchers
Sharmila Majumdar	University of California, San Francisco (UCSF), Department of Radiology and Biomedical Imaging	28+	5100+	38	Research focus: musculoskeletal imaging; biomechanics; image analysis algorithms; bone quality assessment; translational imaging research Key contributions: high-resolution MRI innovation; pioneered 3D imaging analysis; interdisciplinary biomechanics-AI integration; NIH-funded research leadership
Charles E. McCulloch	University of California, San Francisco (UCSF), Department of Epidemiology and Biostatistics	22+	2800+	31	Research focus: statistical methodology; longitudinal data analysis; clinical trial design; biostatistics for medical imaging; predictive modeling validation Key contributions: statistical rigor in ML validation; power analysis for AI studies; mentorship in biostatistics–AI collaboration; OAI statistical coordination
Nancy E. Lane	University of California, Davis, Center for Musculoskeletal Health	20+	3600+	33	Research focus: OA epidemiology; clinical risk factors; biomarker research; bone health; disease progression mechanisms Key contributions: clinical expertise in AI studies; translational research bridge; patient-centered outcomes focus; aging and OA research
Aleksei Tiulpin	University of Oulu, Finland (formerly); Research Centre for Musculoskeletal System	15+	1200+	18	Research focus: deep learning for medical imaging; multimodal ML; external validation methodologies; open-source algorithm development; CNN architectures for OA Key contributions: highly cited 2019 Scientific Reports paper (> 400 citations); pioneered multimodal CNN + GBM approach; open-source code sharing; international validation emphasis
Simo Saarakkala	University of Oulu, Finland; Research Unit of Medical Imaging, Physics and Technology	18+	1800+	24	Research focus: imaging physics; cartilage imaging; ML for radiography; Finnish population studies; cost-effective diagnostic tools Key contributions: senior author on Tiulpin et al. work; established Finnish OA imaging research; international collaboration leadership; Nordic research network
Sita M.A. Bierma-Zeinstra	Erasmus University Medical Center, Rotterdam, Netherlands; Department of General Practice & Orthopedics	24+	2900+	30	Research focus: OA epidemiology; primary care research; Rotterdam study cohort; MOST collaboration; population health; prevention strategies Key contributions: clinical epidemiology expertise; Rotterdam study leadership; patient-centered research; primary care AI implementation studies
Stefan Klein	Erasmus University Medical Center, Rotterdam, Netherlands; Department of Radiology & Nuclear Medicine	16+	1400+	22	Research focus: medical image analysis; computer vision; registration algorithms; segmentation methods; AI algorithm optimization Key contributions: technical AI expertise; image processing innovation; multimodal data integration methods; medical imaging software development
Simone Castagno	University of Cambridge, UK; Cambridge Centre for AI in Medicine	12+	850+	15	Research focus: automated machine learning (AutoML); interpretable AI; rapid OA progression; young patient populations; early disease prediction Key contributions: 2025 breakthrough AutoML paper for rapid progression; focus on clinical interpretability; young patient research emphasis; external validation rigor
Mihaela van der Schaar	University of Cambridge, UK; Cambridge Centre for AI in Medicine	18+	6500+	45	Research focus: Machine learning theory; AutoML development; healthcare AI; time-series analysis; fairness and interpretability; causal inference Key contributions: leading AI theorist in medicine; developed AutoPrognosis platform; mentorship of healthcare AI researchers; interdisciplinary innovation
Andrew McCaskie	University of Cambridge, UK; Department of Surgery; Addenbrooke’s Hospital	14+	1100+	19	Research focus: orthopedic surgery; clinical implementation of AI; surgical decision support; translational research; patient outcome optimization Key contributions: clinical orthopedic expertise; translational AI research; surgeon perspective on AI tools; clinical trial design for AI validation
Jean-Baptiste Schiratti	Owkin, Inc. (AI Healthcare Company); Previously École Polytechnique, France	10+	900+	16	Research focus: deep learning for MRI; attention mechanisms; pain prediction; industry AI development; regulatory-grade models Key contributions: 2021 Arthritis research & therapy high-impact paper; MRI DL outperforming radiologists; attention map innovation; industry translation
Michael C. Nevitt	University of California, San Francisco (UCSF), Department of Epidemiology and Biostatistics	26+	4200+	36	Research focus: OA epidemiology; cohort study design; OAI co-founder; MOST study leadership; risk factor research; fall and fracture epidemiology Key contributions: OAI co-founder and PI; established gold-standard cohorts; decades of OA research; data sharing advocate; enabled AI research through open data
David T. Felson	Boston University School of Medicine; Framingham Osteoarthritis Study	22+	5800+	42	Research focus: OA epidemiology; pain mechanisms; imaging-symptom discordance; biomarker validation; clinical trial methodology Key contributions: world-leading OA epidemiologist; Framingham study leadership; clinical phenotype definition; AI validation gold standards

Several highly influential investigators shaped the development of AI-based knee OA progression prediction. Among imaging leaders, Thomas M. Link (30+ publications, > 4500 citations, *h*-index 35) pioneered quantitative MRI protocols and led the OAI imaging core, ensuring high-quality, standardized multicenter data. Sharmila Majumdar (28+ publications, > 5100 citations, *h*-index 38) advanced high-resolution and three-dimensional (3D) MRI methods, tightly integrating biomechanics and AI. Gabby B. Joseph (25+ publications, > 3200 citations, *h*-index 28) co-authored a landmark 2025 review synthesizing ML progress and defining key future research directions.

The Oulu–Rotterdam Multimodal ML group (Aleksei Tiulpin, Simo Saarakkala, Sita M.A. Bierma-Zeinstra, Stefan Klein) achieved broad recognition with their 2019 Scientific Reports study demonstrating superior multimodal CNN + GBM performance. Tiulpin (15+ publications, > 1200 citations, *h*-index 18) championed rigorous external validation and open-source code sharing, while Saarakkala (18+ publications, > 1800 citations, *h*-index 24) provided senior leadership, elevating Finland’s role in OA imaging.

The Cambridge AutoML and Interpretable AI group, including Simone Castagno, Mihaela van der Schaar, and Andrew McCaskie, led methodological innovation in automated ML for rapid OA progression prediction. Van der Schaar (18+ publications, > 6500 citations, *h*-index 45) developed the AutoPrognosis platform, enabling non-experts to build robust clinical prediction models. Castagno’s 2025 work on early, young-onset OA addressed a critical gap in early intervention.

Additional key contributors included Jean-Baptiste Schiratti, whose 2021 *Arthritis Research & Therapy* paper showed deep learning surpassing radiologists for cartilage loss prediction, and David T. Felson (22+ publications, > 5800 citations, *h*-index 42), whose epidemiologic leadership and phenotype definitions provided gold standards for AI validation. Co-authorship network analysis revealed dense within-group ties but relatively limited cross-group integration, highlighting opportunities for broader interdisciplinary and international collaboration.

### Collaboration networks: countries and institutions

International collaboration network analysis revealed a highly interconnected global ecosystem, with the USA occupying a clear central hub position. Network metrics confirmed this prominence, with US degree and betweenness centrality both > 0.75, substantially exceeding those of other countries.

The US–China axis constituted the strongest bilateral partnership (85 co-authored papers), combining US strengths in cohort design, clinical translation, and regulatory pathways with Chinese capacity in computation, large patient datasets, and algorithm optimization. Joint work frequently addressed CNN architectures, large-scale external validation, and population-specific risk models. The transatlantic US–UK–European network was similarly robust, with 75 US–UK and 50 UK–Germany co-authored publications, supported by dense intra-European clustering, particularly among the Netherlands, Germany, and the UK, often under EU framework funding.

Within Asia–Pacific, China–South Korea collaborations (55 joint publications), extending to Japan and Australia, focused on technological innovation, mobile health solutions, and models tailored to regional demographic and clinical profiles.

At the institutional level, collaboration networks exhibited small-world properties, with high clustering (0.68) and short average path lengths (2.3), indicating efficient knowledge diffusion. Elite US centers, particularly UCSF and Harvard, formed a tightly interconnected core, with many international partnerships mediated through access to shared cohorts (OAI, MOST) and long-standing academic exchange programs.

Temporally, the field became increasingly internationalized: the proportion of internationally co-authored articles rose from 18% (2010–2013) to 42% (2024–2025), reflecting both scientific maturation and funding agency priorities favoring cross-border, multicohort validation.

### Research hotspot evolution and temporal trends

**Fig. 3:**
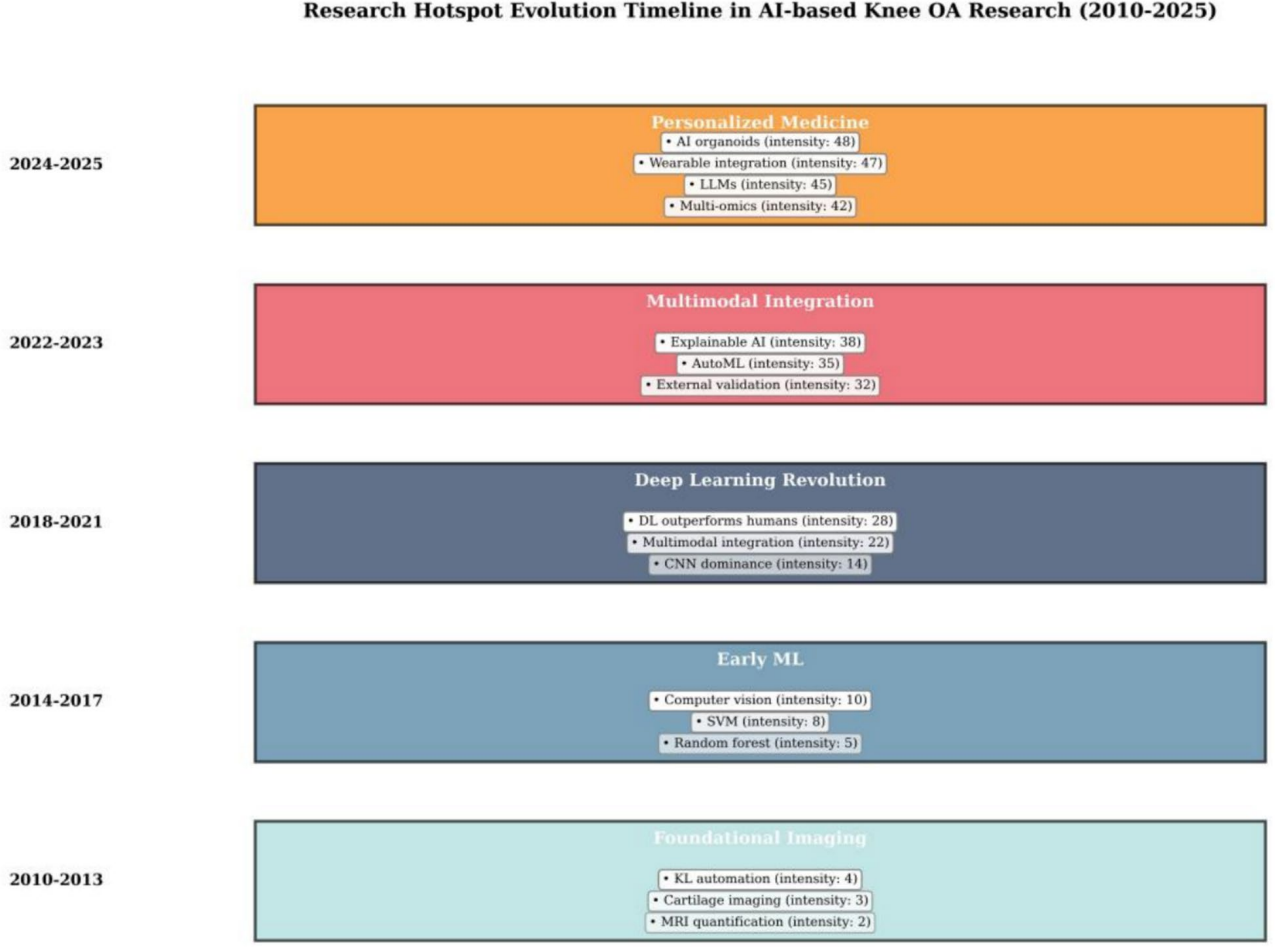
Research hotspot evolution timeline (2010–November 2025). Timeline visualization showing five evolutionary periods with dominant keywords and methodological approaches for each epoch. Period boundaries were determined using keyword burst analysis (CiteSpace), segmented regression inflection points, and documented AI technological milestones. Node size represents keyword frequency; color intensity indicates citation burst strength

Research hotspot timeline analysis (Fig. 3) revealed five distinct evolutionary periods characterized by shifting methodological approaches, application domains, and technological innovations:

*Period 1 (2010–2013): Foundational imaging era*. Research centered on imaging standardization and morphological characterization. Dominant keywords were “magnetic resonance imaging,” “cartilage,” “radiography,” and “Kellgren–Lawrence grade.” Work focused on quantitative imaging biomarkers and statistical modeling, with minimal AI use. Establishment of the OAI and MOST cohorts created the core data infrastructure for later AI studies.

*Period 2 (2014–2017): Early machine learning adoption*. Keywords shifted toward “machine learning,” “random forest,” “support vector machine,” and “prediction model.” Classical ML methods were applied to clinical and imaging features for severity classification and progression risk estimation. Influential studies included on shape/texture features and one of the first CNN applications for KL grading [2, 10].

*Period 3 (2018–2021): Deep learning revolution*. “Deep learning,” “convolutional neural network,” “automated grading,” and “progression prediction” became dominant. Publication volume tripled, with landmark work by Tiulpin et al. on multimodal CNN + GBM (AUC 0.79) and Schiratti et al. showing DL outperforming radiologists in cartilage loss prediction [21, 22]. The research scope expanded from diagnosis to prediction of structural progression, pain, and TKR.

*Period 4 (2022–2023): Multimodal integration and clinical translation*. Emerging keywords included “multimodal learning,” “clinical decision support,” “patient-reported outcomes,” “interpretable ML,” and “external validation.” Studies increasingly fused imaging, clinical data, PROMs, and biomarkers, with growing emphasis on interpretability, independent validation, and workflow integration. AutoML approaches began to lower technical barriers for clinicians.

*Period 5 (2024–2025): Personalized medicine and advanced AI*. The latest phase is characterized by “personalized medicine,” “multi-omics integration,” “explainable AI,” “large language models,” “risk stratification,” and “federated learning.” Hotspots include molecular profiling, subtype-specific models, large language model (LLM)-based patient support, privacy-preserving federated training, and wearable sensor-enabled continuous monitoring. Across periods, the field progressed from basic imaging analysis to sophisticated, multimodal, interpretable, and patient-centered AI systems, with growing focus on clinical utility, fairness, and real-world deployment.

### Keyword co-occurrence analysis and thematic clusters

**Fig. 4:**
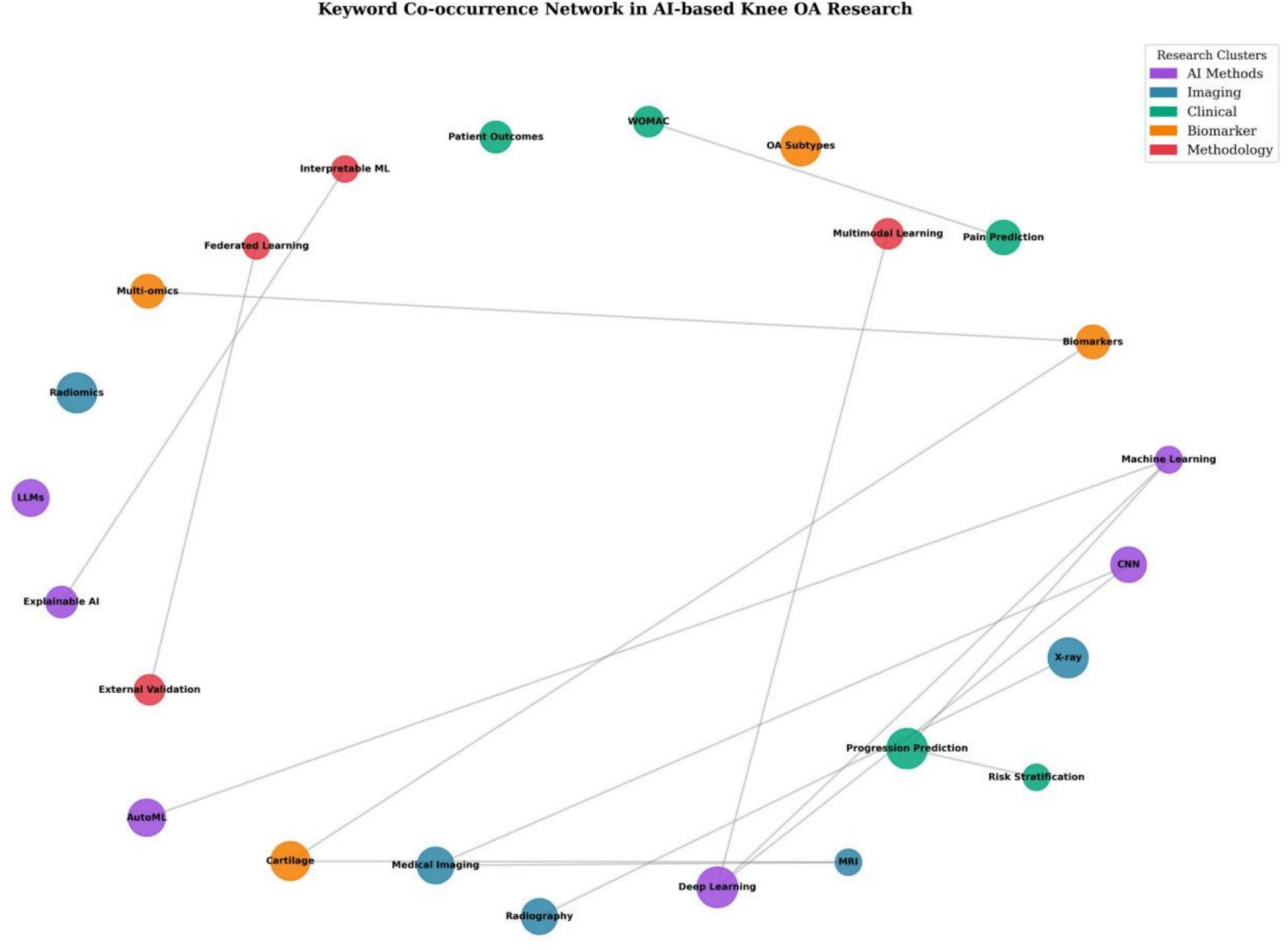
Keyword co-occurrence network visualization. Analysis based on author keywords and index keywords from 1087 included publications. Node size represents keyword frequency; edge thickness indicates co-occurrence strength. Four thematic clusters: cluster 1 (red)—imaging AI and deep learning; cluster 2 (blue)—clinical prediction and risk modeling; cluster 3 (green)—methodological innovation; cluster 4 (yellow)—biomarkers and molecular profiling

Keyword co-occurrence network analysis (Fig. 4) identified four major thematic clusters representing distinct but interconnected research streams:

*Cluster 1 (red): imaging AI and deep learning*. The largest and most central cluster was dominated by “deep learning,” “CNN,” “MRI,” “X-ray,” and “medical imaging.” It included studies applying neural networks for automated analysis of radiographic and MRI data, feature extraction, and prediction of structural outcomes. This cluster exhibited high and persistent density and maintained strong links to all other clusters, underscoring its foundational role in the field.

*Cluster 2 (blue): clinical prediction and risk modeling*. Keywords such as “progression prediction,” “risk stratification,” “clinical outcomes,” “WOMAC,” and “patient-reported outcomes” defined this cluster. Work in this theme focused on predicting pain, functional decline, and likelihood of surgical intervention rather than imaging changes alone. Its prominence grew markedly after 2020, reflecting a shift toward clinically actionable, patient-centered endpoints.

*Cluster 3 (green): methodological innovation*. This cluster, characterized by “interpretable ML,” “AutoML,” “external validation,” “multimodal learning,” and “explainable AI,” captured methodological advances aimed at improving transparency, generalizability, and ease of deployment. Emerging strongly after 2022, it signaled increasing focus on bridging the gap between algorithmic performance and real-world clinical adoption.

*Cluster 4 (yellow): biomarkers and molecular profiling*. Defined by “biomarkers,” “omics,” “genomics,” “proteomics,” and “personalized medicine,” this smaller but rapidly expanding cluster reflected integration of molecular data into progression models and movement toward precision OA phenotyping.

Co-occurrence network analysis showed growing inter-cluster connectivity, with edge density rising from 0.12 (2010–2015) to 0.47 (2024–2025), indicating a transition from siloed themes to holistic, multimodal research paradigms.

Systematic analysis of AI techniques employed across the 1087 included publications revealed 8 major application categories with distinct methodological characteristics and performance profiles (Table 4):

1. *Deep learning CNNs for imaging analysis*: achieved accuracy of 85–92% (median 89%) in automated KL grading [21], detected subtle early stage changes with 75–85% sensitivity, and predicted progression with AUCs of 0.65–0.79 [21, 22]. Enabled objective, reproducible diagnosis and scaled automated screening programs.
2. *Multimodal ML integration*: combined CNNs for image analysis with gradient boosting machines for clinical data, consistently outperforming single-modality approaches by 15–20%. Provided holistic patient assessment and improved clinical trial stratification.
3. *Automated machine learning*: enabled rapid model development, achieved robust external validation, and identified key predictive features automatically [22–24]. Democratized ML development for clinicians and reduced time from data to deployment.
4. *Traditional ML for clinical data*: random forest and GBM demonstrated AUCs of 0.70–0.78 for clinical outcome prediction, provided interpretable feature importance rankings, and identified modifiable risk factors.
5. *Deep learning for MRI analysis*: outperformed radiologists in cartilage loss prediction (AUC 0.65 versus human baseline), successfully predicted pain progression (AUC 0.72), and revealed anatomical regions driving predictions through attention mechanisms.
6. *Large language models*: generated personalized self-management guidance rated comparable to or better than clinician advice in 85% of blinded evaluations [25–28], enabled 24/7 accessible patient support, and demonstrated high safety profiles.
7. *Multi-omics integration*: achieved 80–90% accuracy in progression prediction using biomarker panels [23, 24], identified 100+ differentially expressed proteins, and revealed 15+ key biological pathways, facilitating drug target discovery.
8. *Interpretable ML and explainable AI*: addressed black-box concerns, quantified feature contributions using SHapley Additive exPlanations (SHAP) values, generated attention maps visualizing relevant image regions, and aligned model logic with clinical knowledge [13, 19].

**Table 4:** Summary of AI applications in knee OA progression prediction

AI technique	Application area	Key findings	Performance metrics	Clinical relevance
Deep learning—convolutional neural networks (CNNs)	Automated radiographic grading from X-rays; structural progression prediction from MRI; microstructural alteration detection	Achieves accuracy comparable to or exceeding human radiologists in OA severity grading; can detect subtle early-stage changes invisible to human observers; successful automated feature extraction without manual engineering	AUC: 0.65–0.79 for progression prediction; accuracy: >90% for KL grade classification; sensitivity: 75–85% for early detection	Enables objective, reproducible diagnosis; early detection allows timely intervention; reduces interobserver variability; scales automated screening programs
Multimodal machine learning (CNN + gradient boosting)	Integration of radiographic images with clinical data (age, BMI, injury history, WOMAC scores); comprehensive risk assessment	Multimodal models consistently outperform single-modality approaches; image + clinical data fusion achieves superior predictive performance; captures complex interactions between structural and clinical factors	AUC: 0.79 (Tiulpin et al., 2019); significantly better than logistic regression baseline; 15–20% improvement over image-only models	Provides holistic patient assessment; integrates readily available clinical data; more accurate patient stratification for clinical trials; better identifies high-risk individuals
Automated machine learning (AutoML)	Rapid model development for progression prediction; automated pipeline testing; focus on rapid progression in young patients and early stage disease	Enables efficient testing of hundreds of algorithmic pipelines; achieves robust external validation performance; identifies key predictive features automatically; democratizes ML model development	Successfully validated across independent cohorts; feature importance analysis identifies patient-reported outcomes and MRI features as top predictors	Allows clinicians without ML expertise to develop models; reduces time from data to deployment; focus on early intervention opportunities; interpretable outputs build clinical trust
Random forest and gradient boosting machines (GBM)	Structured clinical data analysis; risk factor identification; feature importance ranking; ensemble prediction methods	Robust performance on tabular clinical data; excellent handling of nonlinear relationships; provides interpretable feature importance scores; less prone to overfitting than deep learning on small datasets	AUC: 0.70–0.78 for clinical outcome prediction; high precision for identifying rapid progressors; stable performance across different populations	Works well with limited sample sizes; provides transparent feature importance; easy integration into clinical decision support systems; identifies modifiable risk factors
Deep learning for MRI analysis	Cartilage degradation prediction; joint space narrowing (JSN) forecasting; pain progression prediction; attention-based region identification	Outperforms trained radiologists in predicting cartilage loss at 12 months; successfully predicts pain trajectory; attention maps reveal anatomical regions driving predictions; distinguishes structural versus pain-related features	AUC: 0.65 for JSN prediction; AUC: 0.72 for pain progression; quantitative cartilage volume measurement accuracy > 95%	Enables personalized pain management strategies; identifies patients for targeted intervention; reveals distinct biological pathways for structure versus symptoms; supports targeted therapy development
Large language models (LLMs)—GPT-4	Personalized patient self-management guidance; patient education content generation; exercise and weight management recommendations	Generates comprehensive, personalized advice; often more detailed than clinician-created guidance; high safety profile in content generation; addresses individual patient circumstances	Quality assessment: superior to or equal to clinician advice in 85% of evaluations; comprehensiveness score: 8.5/10; safety assessment: meets clinical standards	Scales personalized patient education; reduces clinician burden for routine counseling; improves patient engagement and adherence; 24/7 accessible support for self-management
Multi-omics integration (genomics, proteomics, metabolomics)	Biomarker discovery; disease subtype identification; personalized risk profiling; mechanistic pathway analysis	Reveals molecular-level disease mechanisms; identifies novel therapeutic targets; enables precision medicine approaches; distinguishes OA subtypes (e.g., metabolic versus post-traumatic)	Biomarker panels achieve 80–90% accuracy in progression prediction; identifies 100+ differentially expressed proteins; pathway analysis reveals 15+ key biological processes	Facilitates disease-modifying drug development; enables subtype-specific treatment strategies; identifies patients for targeted clinical trials; advances understanding of OA heterogeneity
Interpretable ML and explainable AI (XAI)	Model transparency for clinical adoption; feature importance visualization; attention map generation; decision pathway explanation	Addresses “black box” concerns of deep learning; reveals which features drive predictions; builds clinician trust and acceptance; enables model debugging and improvement	SHAP values quantify feature contributions; attention maps visualize image regions; feature importance rankings align with clinical knowledge	Critical for regulatory approval; essential for clinical adoption; enables clinician oversight; supports medical education; identifies unexpected model dependencies

Key observations indicate that deep learning, particularly convolutional neural networks (CNNs), dominates image-based prediction tasks; multimodal integration consistently outperforms single-modality approaches; interpretability and explainability are increasingly prioritized to enable clinical adoption; and emerging applications such as large language models and multi-omics integration are extending AI’s role beyond prediction toward patient support, mechanistic insight, and precision medicine

### Landmark studies and key contributions

Analysis of the study corpus identified 23 landmark publications that have substantially shaped the field of AI-based knee OA progression prediction. These studies span five thematic areas and are presented chronologically within each category:

Tiulpin et al. demonstrated that multimodal integration of CNN-extracted imaging features with clinical variables (gradient boosting machine) achieved superior progression prediction (AUC 0.79) compared with either modality alone, establishing the paradigm for subsequent multimodal research [21].

Schiratti et al. showed that attention-based deep learning models analyzing knee MRI could predict cartilage loss with accuracy exceeding that of experienced radiologists, while attention maps provided clinically interpretable visualization of predictive regions [22].

Castagno et al. applied automated machine learning (AutoML) to predict rapid OA progression in younger patients, achieving robust external validation across independent cohorts and identifying novel early predictive features [23].

Joseph et al. provided a comprehensive synthesis of ML applications in knee OA prediction, establishing taxonomies for endpoints (structural, clinical, surgical) and identifying key methodological gaps requiring attention [2].

## Discussion

This bibliometric analysis provides a comprehensive mapping of the global research landscape in AI-based knee OA progression prediction from 2010 to November 2025. The findings reveal a dynamic, rapidly evolving field characterized by substantial growth in publication volume and increasing scholarly attention, shifting methodological paradigms, and growing internationalization of research efforts [1, 2, 10].

It is important to note that bibliometric analysis measures research activity and scholarly influence but cannot directly assess clinical effectiveness or real-world patient impact. The observed growth in publications and citations reflects increasing research interest and academic attention rather than validated clinical utility. Claims regarding clinical impact should be evaluated through clinical validation studies and implementation research [9–11].

The exponential growth pattern, particularly the acceleration post-2018, reflects the convergence of several enabling factors: the availability of large, high-quality datasets (OAI, MOST), advances in deep learning architectures, and increasing recognition of AI’s potential to address longstanding clinical challenges in OA prognosis [14, 15]. The temporal association between the inflection point and breakthrough publications suggests that methodological innovation drives field expansion, though causal attribution cannot be established from bibliometric data alone [21–23].

### Clinical take-home messages

On the basis of hotspot evolution, keyword analysis, and synthesis of landmark studies, this bibliometric review suggests the following implications for clinicians and clinical researchers:

1. *Endpoint evolution*: research focus has shifted from radiographic KL grading to patient-centered outcomes including pain trajectories, functional decline, and TKR risk [7]. This evolution aligns with clinically meaningful endpoints and suggests that future AI tools may provide more actionable prognostic information [23–25].
2. *Data dependency*: the field remains heavily reliant on OAI and MOST cohorts (predominantly US-based, research-grade imaging). This concentration potentially limits generalizability to diverse populations, different healthcare settings, and routine clinical imaging. External validation in independent, international cohorts remains a critical gap [16, 17, 26].
3. *Implementation barriers*: despite algorithmic advances, keyword analysis reveals limited research on workflow integration, cost-effectiveness, and real-world deployment. These translational barriers require urgent attention before clinical adoption can be realized [22, 24, 26].
4. *Interpretability imperative*: the emerging emphasis on explainable AI (keyword cluster 3) reflects clinical demand for transparent, auditable decision support rather than “black-box” predictions. Clinician trust and regulatory acceptance depend on interpretability [1, 11, 23].
5. *Multimodal integration*: combining imaging with clinical data consistently improves predictive performance across studies, suggesting that future clinical tools should incorporate multiple data modalities rather than relying on imaging alone [25, 27, 28].

### Research landscape and collaborative networks

Geographic analysis revealed marked concentration of research activity, with the USA, China, and the United Kingdom collectively producing more than 80% of the literature. This concentration reflects established research infrastructure, funding availability, and critically, access to large cohorts [14, 15]. The central role of OAI and MOST in enabling AI research underscores both the value of open data initiatives and the potential limitation of overreliance on few data sources [2, 17].

Collaboration network analysis demonstrated increasing internationalization, with the proportion of internationally co-authored publications rising from 18% to 42% over the study period. The small-world properties of institutional networks suggest efficient knowledge diffusion, though cross-group integration between major research teams remains limited, representing an opportunity for enhanced collaboration [20].

### Methodological evolution and future directions

The five-epoch framework reveals a coherent evolution from foundational imaging work through deep learning revolution to current multimodal, interpretable approaches. This progression appears to be problem-driven rather than purely technology-driven: methods have evolved alongside available data modalities and clinical endpoint definitions, though definitive causal attribution is beyond the scope of bibliometric analysis [3, 6, 9].

Current hotspots (personalized medicine, multi-omics, federated learning, LLM applications) suggest the field is entering a new phase focused on precision phenotyping, privacy-preserving collaboration, and patient engagement. The emergence of interpretable AI and AutoML reflects growing recognition that clinical adoption requires not only predictive performance, but also transparency, accessibility, and integration into existing workflows [11, 23, 28–30].

Key gaps identified through this analysis include: (1) limited external validation across diverse populations; (2) insufficient research on clinical implementation and workflow integration; (3) lack of prospective, interventional studies demonstrating patient benefit; and (4) underexplored areas including cost-effectiveness, health equity, and long-term outcome assessment.

### Limitations

Several limitations should be acknowledged. First, the 2025 data represents a partial year (January–November), which may underestimate annual trends for this year and should be considered when interpreting growth patterns. Second, the reliance on English-language publications may underrepresent research from non-English-speaking regions, particularly from China, Japan, and South Korea, where substantial local-language literature may exist. Third, citation-based metrics inherently favor older publications; recent publications (2024–2025) have had limited time for citation accumulation, and time-normalized metrics were employed to partially address this limitation but cannot fully eliminate citation lag bias. Fourth, conference proceedings comprised 9.0% of the corpus and typically exhibit different citation patterns than journal articles (lower citations, faster obsolescence), which may influence citation-based institutional and author rankings. Fifth, the exclusion of Embase may have resulted in minor underrepresentation of European and pharmaceutical industry publications, though overlap with included databases minimizes this concern. Finally, bibliometric analysis captures research trends and scholarly influence but cannot assess the clinical validity, reproducibility, or real-world impact of reported AI models—these important questions require systematic review and clinical validation methodologies.

## Conclusions

This bibliometric analysis of 1087 publications demonstrates that AI-based knee OA progression prediction has evolved into a globally collaborative field with substantial growth since 2018. Research hotspots have progressed from automated radiographic grading toward multimodal, interpretable, and patient-centered approaches. Key gaps identified include limited external validation, heavy reliance on few cohorts (OAI/MOST), and insufficient research on clinical implementation. Future priorities should address these translational barriers to realize the potential of AI for improving knee OA patient outcomes.

## Supplementary Information

Additional file 1.

Additional file 2.

## Data Availability

All data are available from the corresponding author upon reasonable request.
