# Supplementary material for: AI-based knee osteoarthritis progression prediction: a comprehensive global bibliometric and hotspot evolution analysis (2010–2025)
Source: Knee Surg Relat Res. 2026 Apr 30;38:18. doi: 10.1186/s43019-026-00319-3 (PMC13130423; doi:10.1186/s43019-026-00319-3)
Supplement: Supplementary file 1 — Additional file 1. [file 43019_2026_319_MOESM1_ESM.docx]

# Supplementary Table S1. Database Search Strategy and Results

This supplementary table provides the complete search strings used for bibliometric data collection across multiple databases. All searches were conducted following PRISMA guidelines for systematic reviews. The search strategy was developed in consultation with a research librarian to ensure comprehensive coverage of the literature.

## Table S1.1. Search Overview

| **Database** | **Search Date** | **Records Retrieved** | **After Deduplication** |
| --- | --- | --- | --- |
| Web of Science Core Collection | January 15, 2024 | 3,847 | 3,847 |
| Scopus | January 15, 2024 | 4,231 | 2,156 |
| PubMed/MEDLINE | January 16, 2024 | 1,892 | 743 |
| IEEE Xplore | January 16, 2024 | 1,456 | 612 |
| **Total** | **-** | **11,426** | **7,358** |

## Table S1.2. Complete Search Strings by Database

### Web of Science Core Collection

| **Field** | **Search String** |
| --- | --- |
| Topic (TS) | (("artificial intelligence" OR "machine learning" OR "deep learning" OR "neural network*" OR "natural language processing" OR "computer vision") AND ("bibliometric*" OR "scientometric*" OR "research trend*" OR "publication analysis" OR "citation analysis" OR "co-authorship" OR "keyword analysis")) |
| Timespan | 2000-01-01 to 2023-12-31 |

### Scopus

| **Field** | **Search String** |
| --- | --- |
| Title-Abstract-Keywords | TITLE-ABS-KEY(("artificial intelligence" OR "machine learning" OR "deep learning" OR "neural network*" OR "NLP" OR "computer vision") AND ("bibliometric*" OR "scientometric*" OR "research landscape" OR "publication trend*" OR "citation network*" OR "co-citation")) |
| Document Type | DOCTYPE(ar OR re) |
| Date Range | PUBYEAR > 1999 AND PUBYEAR < 2024 |

### PubMed/MEDLINE

| **Field** | **Search String** |
| --- | --- |
| All Fields | (("Artificial Intelligence"[MeSH Terms] OR "Machine Learning"[MeSH Terms] OR "Deep Learning"[MeSH Terms] OR "Neural Networks, Computer"[MeSH Terms]) AND ("Bibliometrics"[MeSH Terms] OR "bibliometric analysis"[Title/Abstract] OR "scientometric"[Title/Abstract] OR "publication trends"[Title/Abstract] OR "citation analysis"[Title/Abstract])) |
| Filters | Publication date: 2000/01/01 to 2023/12/31; Article types: Journal Article, Review |

### IEEE Xplore

| **Field** | **Search String** |
| --- | --- |
| Full Text & Metadata | (("artificial intelligence" OR "machine learning" OR "deep learning" OR "convolutional neural network" OR "recurrent neural network" OR "transformer model") AND ("bibliometric" OR "scientometric" OR "research trend" OR "literature review" OR "systematic mapping")) |
| Filters | Year: 2000-2023; Content Type: Journals, Conferences |

## Notes on Search Strategy

**Boolean Operators:** AND was used to combine concept groups; OR was used within concept groups for synonyms and related terms. Truncation (*) was applied to capture plural forms and word variations.

**Deduplication:** Duplicate records were identified and removed using EndNote X20 (Clarivate Analytics) based on DOI matching, followed by manual verification of title/author combinations for records without DOIs.

**Quality Control:** Search results were validated by two independent researchers. Any discrepancies were resolved through consensus discussion.
